# A Novel Two-Step Liquid-Liquid Extraction Procedure Combined with Stationary Phase Immobilized Human Serum Albumin for the Chiral Separation of Cetirizine Enantiomers along with M and P Parabens

**DOI:** 10.3390/molecules21121654

**Published:** 2016-12-07

**Authors:** Aleksandra Chmielewska, Lucyna Konieczna, Tomasz Bączek

**Affiliations:** Department of Pharmaceutical Chemistry, Faculty of Pharmacy, Medical University of Gdańsk, Hallera 107, PL-80-416 Gdańsk, Poland; chmola@amg.gda.pl (A.C.); lkon@amg.gda.pl (L.K.)

**Keywords:** enantioseparation, cetirizine enantiomers, human serum albumin (HSA), HPLC determination, pharmaceutical analysis, M and P parabens

## Abstract

The research into the separation of drug enantiomers is closely related to the safety and efficiency of the drugs. The aim of this study was to develop a simple and validated HPLC method to analyze cetirizine enantiomers. In the case of liquid dosage forms, besides the active substance in large amounts there are usually also inactive ingredients such as methyl- and propylparaben. Unfortunately, these compounds can interfere with the analyte, inter alia during chiral separation of the analyte enantiomers. The proposed innovative two-step liquid-liquid extraction procedure allowed for the determination of cetirizine enantiomers (along with M and P parabens) also in liquid dosage forms. The main focus of this study was the chromatographic activity of cetirizine dihydrochloride on the proteinate-based chiral stationary phase. The chromatographic separation of cetirizine enantiomers was performed on an immobilized human serum albumin (HSA) column for the first time. Measurements were performed at a wavelength of 227 nm. Under optimal conditions, baseline separation of two enantiomers was obtained with 1.43 enantioseparation factor (α) and 1.82 resolution (*R*_s_). Finally, the proposed method was successfully applied to the selected pharmaceutical formulations.

## 1. Introduction

Cetirizine is a second-generation anti-histamine, carboxylated metabolite of hydroxyzine, indicated for the treatment of seasonal allergic rhinitis, perennial allergic rhinitis and chronic idiopathic urticarial. Cetirizine has two enantiomers due to the inclusion of one chiral center in the chemical structure, and these enantiomers have different pharmacological effects. Levocetirizine (R-form) is the active enantiomer and has a potent anti-histamine effect. It has a two-fold higher affinity for the H_1_-receptor than does the racemic mixture and a 30-fold higher affinity than is seen for dextrocetirizine (S-form) [1].

It is currently marketed as a racemate. Cetirizine dihydrochloride can be found in a range of pharmaceutical forms such as tablets, compounded capsules, syrups, drops and oral liquids [2,3,4,5,6,7]. The tablets are more preferred over the oral liquid formulation. In addition, oral liquids require the presence of antimicrobial agents such as parabens. Parabens are a homologous series of preservatives commonly used in the food, cosmetic and pharmaceutical industries. They are derivatives of *p*-hydroxybenzoic acid esterified at the C-4 position. Parabens are effective in a wide pH range and present a broad spectrum of antimicrobial activity, although they are the most effective against yeast and molds [8]. Generally, methylparaben (paraben M) and propylparaben (paraben P), and sometimes butylparaben are used for the preservation of various liquid dosage forms with cetirizine [5,6].

There are available reports of several chromatography methods developed for quality control of racemic cetirizine in biological samples and pharmaceutical formulations. Some authors reported that enantiomers of cetirizine were separated by means of HPLC-UV, LC–mass spectrometry and supercritical fluid chromatography with α_1_-acid glycoprotein or ovomucoid protein based chiral stationary phase [9,10,11,12] and polysaccharide-derived chiral stationary phase [13,14,15,16,17]. In addition, the capillary electrophoresis method was used for the chiral separation of cetirizine enantiomers in biological fluids samples and tablets. In this case, chiral separation was carried out using sulfated-β-cyclodextrins as selectors [7,18,19,20]. Moreover, branched and linear polysaccharides (glycogen and amylose or maltodextrin) [21,22,23] and macrocyclic antibiotics [24,25,26] were also used as selectors to obtain a baseline separation for racemic cetirizine.

However, none of the cited chiral methods were used in the determination of cetirizine enantiomers along with inactive ingredients such as M and P parabens in a liquid dosage form. Currently, pharmaceutical companies commonly declare the presence and quantitative contents of parabens in liquid forms of this drug. It should be noted that the inspection of cetirizine and listed parabens in syrup, drops and oral solution was only conducted in achiral conditions using chromatographic [5] and electrophoretic [6] methods.

The aim of this work was to develop a simple HPLC method for chiral separation of cetirizine and simultaneous determination of M and P parabens in liquid dosage forms. At the time of method development, no literature data were available on simultaneous determination of cetirizine enantiomers and those inactive ingredients on an immobilized human serum albumin column. However, the revealed interference between paraben M and (*S*)-cetirizine was the reason for the development of the extraction procedure of analytes. The proposed innovative two-step liquid-liquid extraction procedure allowed the separation of the cetirizine enantiomers and parabens in a liquid dosage forms prior to chromatographic analysis. The optimized method was then validated for the assays of cetirizine enantiomers and parabens in drops and oral solution.

## 2. Results and Discussion

### 2.1. Chromatographic Conditions and Selectivity

**Figure 1:**
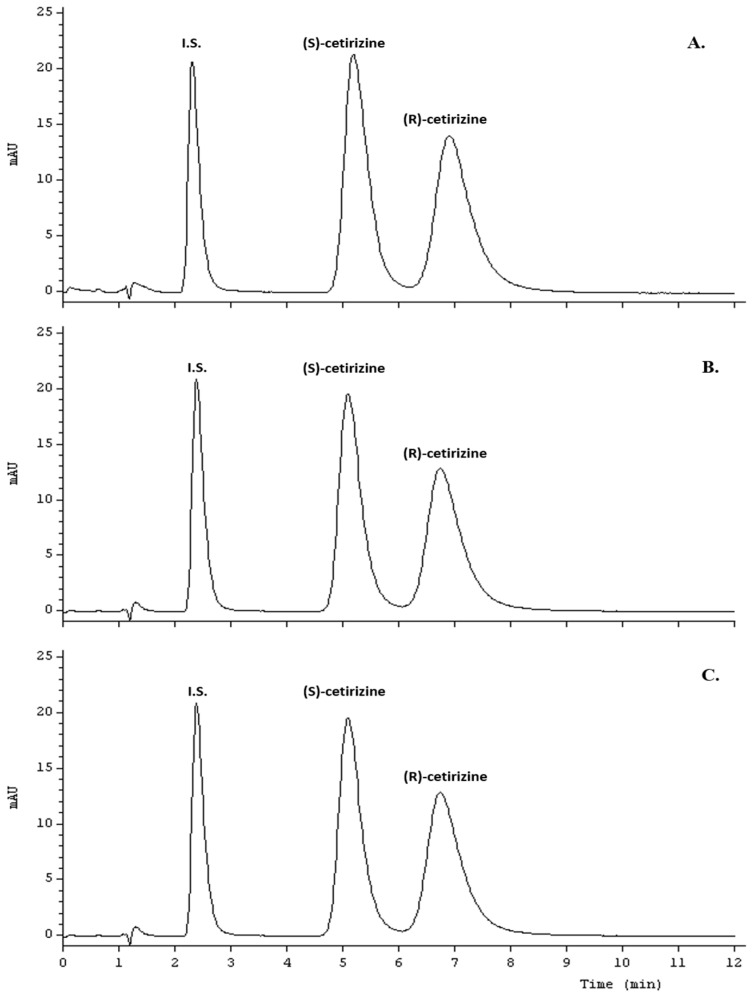
Representative chromatograms of chiral separation of cetirizine enantiomers: (**A**) standard solution containing 10 µg·mL^−1^ of (*R*)-cetirizine (levocetirizine); (**B**) standard solution containing 20 µg·mL^−1^ of racemic cetirizine and 2 µg·mL^−1^ of moclobemide as an internal standard (I.S.); (**C**) chromatogram for ZYRTEC^®^ 10 mg tablets.

The chiral separation of racemic cetirizine was first conducted with the use of an immobilized human serum albumin (HSA) column and binary mobile phases consisting 2-propanol and Sorensen’s phosphate buffer at pH 7.0. So far, immobilized α_1_-acid glycoprotein (AGP) [9,10,11] and ovomucoid (OVM) [12] were used as a protein chiral stationary phase in the separation of cetirizine enantiomers. All papers described above concern chiral separation of cetirizine in biological samples. In this case, the mobile phase was a mixture of acetonitrile and phosphate buffer (pH 7) [9,10] or acetate buffer (pH 7 or 6.6) [11,12]. Only Choi et al. [9] presented resolution and enantioselectivity factor of cetirizine enantiomers as follow *R*_s_ = 2.0 and α = 1.98, however with long analysis time exceeds to 30 min. Chiral separation of this analyte was performed on other type of polysaccharide stationary phases (Chiralcel OD-R [13], Chiralpak AD-H [14,15], Chiralpak IE [17]). The obtained results of the resolution and enantioselectivity factor were *R*_s_ = 1.66 and α = 1.29 [13]; *R*_s_ = 1.85 and α = 1.68 [15]; *R*_s_ = 2.1 and α, not given [17]. In our investigation the best separation of the two enantiomers of cetirizine and moclobemide (I.S.) was achieved with the use of a CHIRALPAK^®^ HSA column and mobile phase 2-propanol—10 mM phosphate buffer pH 7 (10:90 *v*/*v*) at 227 nm. An optimal flow-rate was 0.9 mL·min^−1^ at 25 °C (see Table 1). Optimal retention times were 5.08 min and 6.73 min for (*S*)- and (*R*)-cetirizine, while for moclobemide (I.S.) 2.38 min. The total time of analysis with the regeneration of the column was 12 min. Good chiral separation of the racemic cetirizine was achieved with enantioselectivity factor of α = 1.43 and resolution *R*_s_ = 1.82 (see Figure 1). Finally, the obtained by us chromatographic parameters of chiral separation of cetirizine enantiomers confirm that the developed method could be an alternative to literature methods using other chiral protein stationary phases [9,10,11,12].

The developed method was to be subjected to statistical analysis and used for the determination of enantiomers of cetirizine not only in tablets, but also in drops and oral solution. Having established the active ingredient in the liquid form of the drug, the manufacturer also declares the presence of excipients hence, an imminent possibility of interference in the chromatographic image should be put into consideration. Paraben M (1.35 mg·mL^−1^), paraben P (0.15 mg·mL^−1^) and sodium saccharin (quantity not declared) are pharmacologically inactive ingredients present in the drops (ZYRTEC^®^ 10 mg·mL^−1^) and oral solution (ZYRTEC^®^ 1 mg·mL^−1^). Moreover, d-sorbitol (450 mg·mL^−1^) is present in the oral solution. The chromatographic analysis was accordingly conducted to evaluate the standards of these substances in the optimal conditions of chiral separation of the enantiomers of cetirizine. The liquid dosage forms after dilution also were chromatographically analyzed. In that case, the time of chromatographic analysis was extended to 20 min.

**Figure 2:**
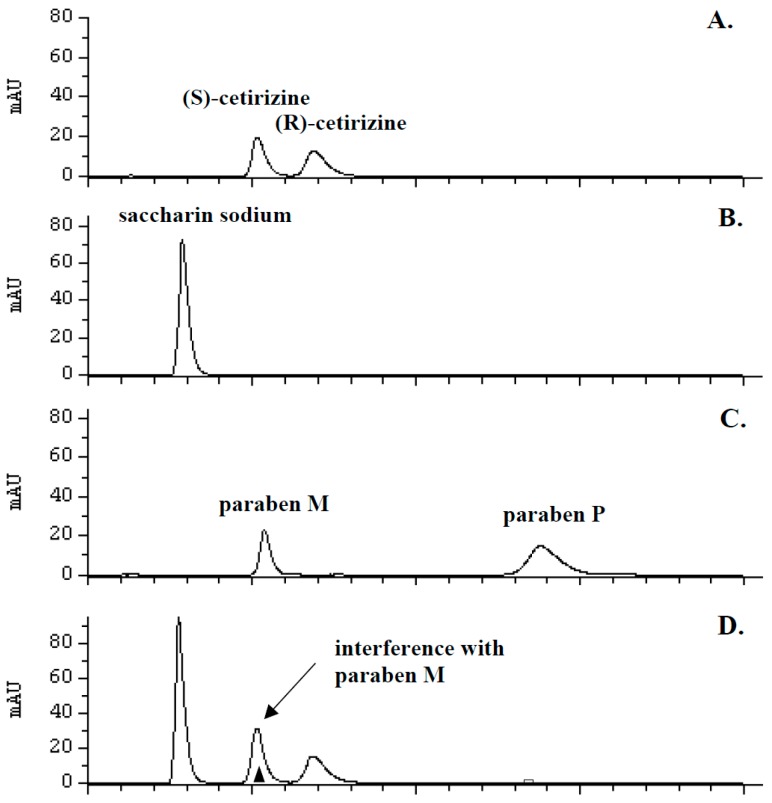

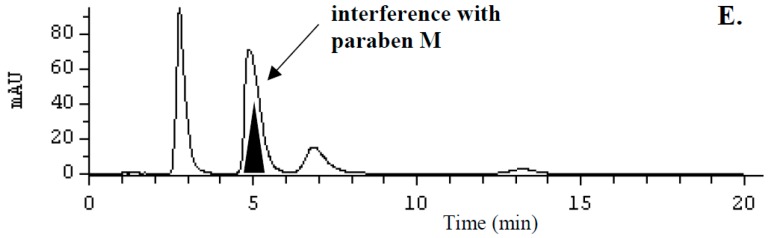
Typical chromatograms showing separation of: (**A**) standard solutions of racemic cetirizine (20 µg·mL^−1^); (**B**) standard solution of saccharium sodium (20 µg·mL^−1^); (**C**) mixture of standard solution of paraben M (10 μg·mL^−1^) and paraben P (20 μg·mL^−1^); (**D**) Zyrtec^®^ 10 mg·mL^−1^ oral drops after direct dilution; (**E**) Zyrtec^®^ 1 mg·mL^−1^ oral solution after direct dilution.

The chromatographic analysis of the drops and oral solution after direct dilution confirmed the presence of the interference between peaks of (*S*)-cetirizine and paraben M. In the case of sodium saccharin and paraben P, the peaks of both compounds are clearly separated from the peaks of the enantiomers of cetirizine. Under the described conditions of the chromatographic analysis, d-sorbitol showed no detection. The results are presented in Figure 2. Due to certain limitations associated with the nature of the protein stationary phase, it was not possible to introduce too radical changes in the conditions of the chromatography. Several parameters of the analysis of isocratic elution were altered in order to improve of the separation of this interfering peaks. Table 1 summarizes the effect of different organic solvents and buffers concentration on the chromatographic parameters. However, applied changes did not produce the desired effect. Therefore, in this case, a decision was made to develop the extraction procedure to eliminate paraben M from the sample prior to chromatographic analysis.

### 2.2. Optimization of Sample Preparation

The sample preparation procedure was not complicated in the case of tablets. It proceeded in a standard way. The representative chromatogram is presented in Figure 1C. The procedure, however, is completely different in the case of the samples of liquid dosage forms.

On the chromatograms for the liquid dosage forms after direct dilution was found the interference between of (*S*)-cetirizine and paraben M. In order to separate the two compounds a liquid-liquid extraction procedure for sample of drops and oral solution is necessary prior to the chromatographic analysis. A number of extractions were conducted with the use of several organic solvents. Certain previously developed methods for the assays of cetirizine in the liquid dosage form were also recommendable in solvent selection: spectrophotometry [27,28,29], chromatography [3,4,5] or capillary electrophoresis [6]. The liquid-liquid extraction was the most frequently used one. However, not all of the cited methods take into account the fact that, besides cetirizine, these pharmaceutical formulations contain parabens [3,4,30]. It should be emphasized that these methods are adequate for the assays of racemic cetirizine in achiral conditions. Nevertheless, preservatives present in liquid dosage forms can sometimes interfere during the evaluation of an active agent. Especially if the assay relates to chiral compounds.

Chloroform [27,28,29] was tested, but the results indicated that this organic solvent was not suitable for the selective extraction of analyzed substances. We also tested dichloromethane and ethyl acetate. These organic solvents were frequently used for the isolation of racemic cetirizine from plasma or urine samples [9,10,14,18,31]. Further research confirmed that dichloromethane in a neutral medium was more effective than ethyl acetate in relation to cetirizine. Additionally, diethyl ether was tested but the obtained results indicated that the extraction of cetirizine was not effective. However, this organic solvent in an alkaline medium was the most appropriate for the extraction of the preservatives (M and P parabens).

The end of the extraction procedure was defined in the analysis of individual extraction variants. As a result, preparing liquid dosage forms samples for racemic cetirizine and parabens analysis was based on a two-step liquid–liquid technique: Step I with diethyl ether after the addition of 1 M NaHCO_3_; Step II with dichloromethane after the addition of 1 M HCl. In these conditions ethyl ether was reliable only for selective parabens extraction. In our study it was observed that 1 M NaHCO_3_ is more suitable than 1 M NaOH for the alkanization of these samples. Hydrochloric acid (Step II) was added to the aqueous layer from Step I of the extraction to neutralize the residue of sodium bicarbonate. Dichloromethane was chosen for Step II of the extraction procedure because under these conditions only cetirizine was obtained. Furthermore, the recoveries of racemic cetirizine were higher than it was the case with organic solvents. The proposed sample preparation was selective and efficient. Saccharin sodium remained in the aqueous layer in the optimised conditions of the two-step extraction procedure. In the case of this compound, this stage does not proceed quantitatively. Thus, the obtained chromatograms do not show the peak of sodium saccharin.

**Figure 3:**
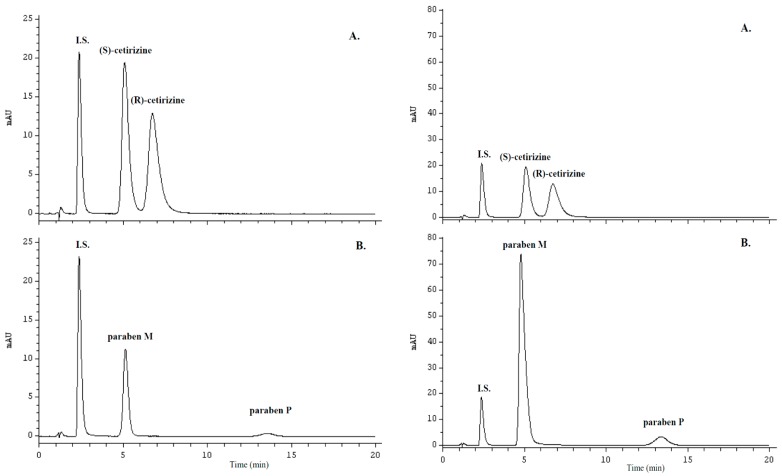
Representative chromatograms for ZYRTEC^®^ 10 mg·mL^−1^ oral drops (on the **left**) and for ZYRTEC^®^ 1 mg·mL^−1^ oral solution (on the **right**); (**A**) cetirizine enantiomers and moclobemide as an internal standard (Step II of the two-step liquid-liquid extraction procedure); (**B**) M and P parabens with moclobemide as an internal standard (Step I of the two-step liquid-liquid extraction procedure).

The results of the two-step liquid-liquid extraction procedure are presented in Figure 3.

### 2.3. Response Function and Linearity

A linear response was obtained for both (*S*)- and (*R*)-cetirizine for direct determination in tablets and likewise, in drops or oral solution after two-step liquid-liquid extraction. Complete response function parameters can be found in Table 2. The absorption maximum of cetirizine was determined at 227 nm. For quantitative determination of (*S*)- and (*R*)-cetirizine linear calibration curves were obtained in the range of 0.25–25.0 μg·mL^−1^.

Furthermore, linear responses were obtained for M and P parabens for drops and oral solution assays after two-step liquid-liquid extraction. Complete response function parameters can be found in Table 2. For quantitative determination of paraben M and paraben P linear calibration curves were obtained in the range of 0.45–54.0 μg·mL^−1^ and 0.05–6.0 μg·mL^−1^, respectively.

Calibration curve equations were obtained in the least squares linear regression analysis of the peak area ratios of each analyte to moclobemide as an internal standard.

### 2.4. Precision and Accuracy

The within-day and between-day precision values for analytes are summarized in Table 3.

Recoveries were calculated as the percentage of each analyte response (peak areas) after the sample preparation procedure mentioned above and compared to that of non-extracted samples containing the analyte at a corresponding concentration of six independently made replications at three concentration levels, i.e., 2.5, 10.0 and 20.0 µg·mL^−1^ for (*S*)-cetirizine and (*R*)-cetirizine and at 2.7, 27.0 and 45.0 µg·mL^−1^ for paraben M and 0.3, 3.0 and 5.0 µg·mL^−1^ for paraben P. The results obtained in the accuracy study are presented in Table 3.

### 2.5. Application of the Validated Method to Pharmaceutical Products

The validated method was successfully used for the enantioselective assay of cetirizine in various commercial pharmaceutical preparations, as given in Table 4. The enantiomers of cetirizine and M and P parabens in drops and oral solution were determined after innovative two-step liquid-liquid extraction procedure. The representative chromatograms for tablets, drops and oral solution are shown in Figure 1C and Figure 3), respectively. No interferences from the sample solvent, impurities, and excipients were detected in the separation of cetirizine enantiomers, parabens and moclobemide as an internal standard at a 227 nm detection wavelength. Furthermore, the method successfully resolved for two preservatives, M and P parabens used in liquid dosage forms (see Figure 3). These two preservatives can be quantified if their quantities are declared.

The mean percentage recoveries of cetirizine enantiomers, M and P parabens obtained after three repeated experiments were found between 98.00% and 99.83%; 98.52% and 99.26%; 98.00% and 98.77%, respectively (Table 4), indicating that the results are accurate and precise. The chiral method permitted determination of the enantiomeric ratio of cetirizine in the preparations. The results in Table 4 indicate that the drug was present as a racemate.

## 3. Experimental Section

### 3.1. Reagents

The standards of racemic cetirizine dihydrochloride, levocetirizine dihydrochloride, paraben M (methyl 4-hydroxybenzoate) and paraben P (propyl 4-hydroxybenzoate) and saccharin sodium were obtained from Sigma-Aldrich (Saint Louis, MO, USA). Moclobemide, used an internal standard (I.S.) was provided by Biovena Pharma (Warsaw, Poland). Dichloromethane, methanol LiChrosolv^®^ and 2-propanol LiChrosolv^®^ were purchased from Merck (Darmstadt, Germany). Ethyl ether, hydrochloric acid, D-sorbitol, sodium bicarbonate, sodium nitrate, di-sodium hydrogen phosphate and sodium dihydrogen phosphate were procured from POCH (Gliwice, Poland). Ultrapure water was obtained from Milli-Q apparatus (Millipore, Bedford, MA, USA).

### 3.2. Instrumentation

The chromatographic system consisted of the following components which all were obtained from Knauer (Berlin, Germany). Namely, solvent pump (Mini-Star K-500), column thermostat jet stream 2 plus with injection valve (20 µL loop) (D-14163), a variable wavelength UV detector (K-2500) and a computer system for data acquisition (Eurochrom 3.05, Wissenschaftliche Gerätebau Dr. Ing. Herbert Knauer GmbH, Berlin, Germany).

### 3.3. Preparation of Stock and Standard Solutions

Stock solutions (1 mg·mL^−1^) of racemic cetirizine dihydrochloride, levocetirizine dihydrochloride, saccharin sodium and d-sorbitol were prepared in water. Stock solutions (1 mg·mL^−1^) of moclobemide (I.S.) and M and P parabens were prepared in methanol. These solutions were stored in the dark under refrigeration at 4 °C. The stability of the standard solutions was checked periodically by injecting a solution of the analyte. Standard working solutions of all analyzed standard substances were prepared by means of diluting stock solutions with mixture of 2-propanol and water at the proportion of 10:90 (*v*/*v*).

### 3.4. Sample Preparation

#### 3.4.1. Tablets

Ten cetirizine tablets (label claim: 10 mg of racemic cetirizine dihydrochloride/tablet) were accurately weighed, their mean weight were determined, and they were then finely powdered. An amount equivalent to 10 mg was transferred into a 50 mL volumetric flask, 30 mL of water was added, then the substance was sonicated for 15 min, diluted with water to 50 mL and a 5 mL sample taken from this solution was centrifuged at 9000 rpm for 15 min. Then a 3-mL aliquot from supernatant was decanted to another test tube and was centrifuged again at 12,000 rpm for 5 min. A 100 µL aliquot was transferred into the *Eppendorf*, then 20 µL of the internal standard (100 µg·mL^−1^) was added and diluted to 1 mL with the mobile phase. 20 µL of the prepared sample was injected into the chromatographic system. The amounts of (*S*)- and (*R*)-cetirizine were individually calculated for particular dosage forms based on related linear regression equations.

#### 3.4.2. Drops and Oral Solution

Before starting of the extraction procedure, liquid forms of the drug were diluted: 200 µL of drops (10 mg·mL^−1^ of racemic cetirizine dihydrochloride) was transferred into a 20 mL volumetric flask and diluted with water to 20 mL. Similarly, 1 mL of oral solution (1 mg·mL^−1^ of racemic cetirizine dihydrochloride) was transferred into 10 mL volumetric flask and diluted to 10 mL with water. In both cases, racemic cetirizine solution was obtained and its approximate concentration was 100 µg·mL^−1^. Then, 1 mL of this solution was subjected to the two-step extraction procedure.

Step I

1 mL of this solution was transferred into a 7 mL the centrifuge test tube. Extraction was performed by adding 0.5 mL 1 M NaHCO_3_ and 3 mL diethyl ether. The next steps were in each case: vortex-mixing for 60 s and shaking the contents of the tube mechanically for 30 min at 240/4 amplitude. After 10 min of centrifugation at 3000 rpm the ether layer was transferred quantitatively to another clean glass test tube and evaporated to dryness at 40 °C under vacuum conditions with the use of a CentriVap (Labconco, Kansas City, MO, USA). The residue was reconstituted in 1 mL 10 mM phosphate buffer; 200 µL aliquot was transferred into the Eppendorf, then 20 µL of the internal standard (100 µg·mL^−1^) was added and diluted to 1 mL with the mobile phase. After 7 min of centrifugation at 9000 rpm 20 µL, the prepared sample was injected into the chromatographic system. The amounts of M and P parabens were individually calculated in particular dosage forms with the use of related linear regression equations.

Step II

0.5 mL of 1 M HCl was added to the aqueous layer (from Step I), the contents of effervescence ceases were vortex-mixed, then 2.5 mL of dichloromethane was added. The mixture was shaken mechanically for 30 min at 240/4 amplitude. After 10 min of centrifugation at 3000 rpm, the organic layer was separated, transferred to a clean glass test tube and evaporated to dryness at 40 °C under vacuum conditions with the use of a CentriVap (Labconco, Kansas City, MO, USA). The residue was reconstituted in 1 mL 10 mM phosphate buffer; a 200 µL aliquot was transferred into the *Eppendorf*, then 20 µL of internal standard (100 µg·mL^−1^) was added and diluted to 1 mL with the mobile phase. After 7 min of centrifugation at 9000 rpm, 20 µL of the prepared sample was injected into the chromatographic system. The amounts of cetirizine enantiomers were individually calculated for particular dosage forms with the use of related linear regression equations.

### 3.5. Chromatographic Conditions

Chromatographic separation was conducted with the use of CHIRALPAK^®^ HSA (5 µm particle size, 100 × 4 mm id.). The HPLC analytical column was obtained from Chiral Technologies (Illkirch, France). A binary mixture of 2-propanol and Sorensen’s phosphate buffer at pH 7.0 was applied as the mobile phase. In this experiment, the mobile phase was system: 2-propanol—10 mM phosphate buffer pH 7 (10: 90 *v*/*v*). The flow-rate of the mobile phase was 0.9 mL·min^−1^. The monitoring wavelength was 227 nm and peak areas were used. Chromatograms were performed at 25 °C. In this study, chromatographic retention factors *k* were determined with NaNO_3_ peak as a marker of a column dead volume.

pH value of Sorensen’s phosphate buffer was measured before the preparing mobile phases. pH value was measured with pH meter (Beckman Instruments, Fullerton, CA, USA). The mobile phases were vacuum-filtered through a 0.45 μm membrane filter and degassed in an ultrasonic bath before use.

### 3.6. Validation of the Analytical Method

The method was validated in compliance with the International Conference on Harmonization (ICH) guidelines [32].

#### 3.6.1. Linearity

Calibration curves were constructed by plotting peak area ratios (cetirizine/internal standard) of the standards versus concentration at the eleven concentration levels. Each concentration level underwent six-fold preparation. The calibration curves for both cetirizine enantiomers exhibited linearity within the range of 0.25–25.0 μg·mL^−1^.

Similarly, calibration curves were constructed for M and P parabens. The calibration curves for paraben M and paraben P exhibited linearity within the 0.45–54.0 μg·mL^−1^ and 0.05–6.0 μg·mL^−1^ range, respectively.

#### 3.6.2. Sensitivity

The sensitivity of the method was determined with respect to LOD and LOQ. A calibration curve of racemic cetirizine and M and P parabens were prepared within the studied concentration ranges with the of a mixture of 2-propanol and water at the proportion of 10:90 (*v*/*v*). The limits of detection and quantitation were calculated according to the ICH guidelines [9]. For each analyte, the limit of detection (LOD) was calculated from six independently made replications, and was estimated as the lowest detectable of the sample concentration at which the peak was calculated—three times that of the baseline noise. The limit of quantification (LOQ) was defined as the lowest concentration which can be quantified with the precision expressed by relative standard deviations (RSD %) below 15%, accuracy expressed as the percentage of the nominal concentrations within 80%–120%, and the signal-to-noise ratio better than 10.

#### 3.6.3. Precision

The assays were repeated three times within the same day to obtain method repeatability (intraday precision) and three times over three different days to obtain intermediate precision (interday precision). Both values were expressed as RSD %.

#### 3.6.4. Accuracy

The recovery method was studied with the use of quality control standards prepared at low, middle, and high concentration level. At each level, samples were prepared six times and analyzed according to the previously described procedure. Calculations were performed of the amount of each analyte recovered in relation to the added amount (recovery percent).

## 4. Conclusions

For the first time, effective enantioseparation of cetirizine was successfully completed by means of HPLC with stationary phase immobilized human serum albumin. The proposed method, developed and validated for the determination of racemic cetirizine in tablets, drops and oral solution. The method described here represents a simple, rapid, and efficient analytical method for enantioseparation of this drug in solid dosage forms.

What is important, the proposed HPLC method employing an innovative two-step liquid-liquid extraction procedure for sample preparation is reliable for the determination of racemic cetirizine, M and P parabens in oral pharmaceutical solutions. The method also provides an efficient clean-up procedure of the pharmaceutical formulation. The method was validated and the obtained results were within acceptable limits. Method validation for quantitation of cetirizine enantiomers and M and P parabens showed that the method offers high accuracy. The method can be successfully applied for the identification, quantitative analysis of cetirizine enantiomers and parabens in liquid pharmaceutical formulations.

## Figures and Tables

**Table 1:** The chromatographic parameters of chiral separation of cetirizine enantiomers along with paraben M under various conditions on CHIRAL-HSA column.

Mobile Phase					Retention Factor *k*		Enantioselectivity Factor α	Resolution Factor *R*_s_
Organic Solvent (A)	PB ^a^ mM pH 7 (B)	A:B (*v*/*v*)	Flow-Rate (mL·min^−1^)	Temp. (°C)	PM ^b^	(*S*)-Cetirizine		
2-propanol	10	14:86	0.9	25	2.72	2.65	1.10	0.80
2-propanol	10	10:90	0.9	25	3.27	3.23	1.43	1.82
			0.7	25	4.85	4.75	1.32	1.62
			0.5	25	5.78	5.85	1.20	1.40
			0.9	18	5.10	4.98	1.30	1.45
2-propanol	10	6:94	0.9	25	5.75	5.82	1.21	1.54
2-propanol	20	6:94	0.9	25	5.65	5.61	1.25	1.59
acetonitrile	10	10:90	0.9	25	3.05	3.10	1.15	0.65
acetonitrile	10	6:94	0.9	25	3.82	3.90	1.26	1.12
acetonitrile	20	6:94	0.9	25	3.78	3.77	1.25	1.10
acetonitrile	10	5:94		25	5.80	5.90	1.38	1.65
			0.9	18	7.45	7.54	1.28	1.38
acetonitrile	10	4:96	0.9	25	6.88	6.75	1.32	1.45
acetonitrile	20	4:96	0.9	25	6.70	6.62	1.38	1.48

^a^ PB—phosphate buffer; ^b^ PM—paraben M.

**Table 2:** Validation of analytical method for the assays of cetirizine enantiomers and parabens in pharmaceutical formulations.

	Tablets		Oral Drops or Oral Solution			
Parameter	(*S*)-Cetirizine	(*R*)-Cetirizine	(*S*)-Cetirizine	(*R*)-Cetirizine	Paraben M	Paraben P
Linearity range, µg·mL^−1^	0.25–25	0.25–25	0.25–25	0.25–25	0.45–54	0.05–6
LOD, µg·mL^−1^ ^(a)^	0.1	0.1	0.1	0.1	0.02	0.02
LOQ, µg·mL^−1^ ^(b)^	0.25	0.25	0.25	0.25	0.05	0.05
Slope (±SD)	0.207 (±0.002)	0.207 (±0.002)	0.204 (±0.003)	0.205 (±0.003)	0.190 (±0.002)	0.166 (±0.002)
Intercept (±SD)	0.031 (±0.023)	0.032 (±0.025)	0.024 (±0.036)	0.023 (±0.034)	0.006 (±0.047)	0.0008 (±0.005)
Correlation coefficient, *R* (*R*^2^)	0.9996 (0.9992)	0.9996 (0.9991)	0.9990 (0.9981)	0.9991 (0.9983)	0.9997 (0.9993)	0.9995 (0.9991)
Standard error of the regression (s*_y_*_/*x*_)	0.05	0.05	0.08	0.07	0.10	0.01
Number of data points (*n*)	11	11	11	11	10	10

^(a)^ LOD—limit of detection; ^(b)^ LOQ—limit of quantitation.

**Table 3:** Intra-assay precision, inter-day precision, and recovery at three concentration levels for analytes.

Nominal Concentration (μg·mL^−1^)	Intra-Day Precision (*n* = 6)		Inter-Day Precision (*n* = 6)		Accuracy as Recovery (%)
	Concentration Found (±SD) (μg·mL^−1^)	RSD (%)	Concentration Found (±SD) (μg·mL^−1^)	RSD (%)	
**for direct determination in tablets**					
**(*S*)-cetirizine**					
**2.5**	2.50 ± 0.16	6.63	2.54 ± 0.18	7.15	99.84
**10**	10.00 ± 0.33	3.30	10.25 ± 0.37	3.58	100.00
**20**	20.25 ± 0.25	1.24	20.52 ± 0.30	1.35	101.23
**(*R*)-cetirizine**					
**2.5**	2.49 ± 0.16	6.58	2.53 ± 0.18	7.18	99.72
**10**	10.00 ± 0.32	3.25	10.25 ± 0.37	3.55	100.01
**20**	20.25 ± 0.25	1.23	20.53 ± 0.29	1.33	101.25
**for determination after two-step liquid-liquid extraction from oral drops or oral solution**					
**(*S*)-cetirizine**					
**2.5**	2.49 ± 0.17	6.79	2.53 ± 0.19	7.05	99.59
**10**	9.72 ± 0.34	3.48	9.83 ± 0.36	3.52	97.16
**20**	20.07 ± 0.27	1.32	20.10 ± 0.30	1.24	100.37
**(*R*)-cetirizine**					
**2.5**	2.49 ± 0.17	6.74	2.52 ± 0.19	6.96	99.69
**10**	9.75 ± 0.34	3.46	9.83 ± 0.37	3.55	97.47
**20**	20.10 ± 0.27	1.30	20.11 ± 0.29	1.25	100.48
**Paraben M**					
**2.7**	2.63 ± 0.19	7.23	2.68 ± 0.20	7.58	97.58
**27**	26.50 ± 0.81	3.06	27.22 ± 0.92	3.42	98.15
**45**	44.37 ± 0.68	1.53	44.83 ± 0.79	1.42	98.60
**Paraben P**					
**0.3**	0.29 ± 0.02	7.38	0.32 ± 0.03	7.68	97.07
**3.0**	3.00 ± 0.10	3.37	3.11 ± 0.14	3.56	99.92
**5.0**	5.04 ± 0.08	1.60	5.09 ± 0.10	1.50	100.86

**Table 4:** Analysis of cetirizine commercial pharmaceutical preparations.

Pharmaceutical Preparation ^x^	Label Claim	Recovery ± SD (%) ^y^			
		(*S*)-Cetirizine	(*R*)-Cetirizine	Paraben M	Paraben P
ZYRTEC^®^ coated tablets	10 mg per tablet	99.80 ± 0.53	99.83 ± 0.55	n.d. ^z^	n.d. ^z^
ZYRTEC^®^ drops	10 mg·mL^−1^	98.65 ± 0.81	98.68 ± 0.83	98.52 ± 0.74	98.00 ± 0.85
ZYRTEC^®^ oral solution	1 mg·mL^−1^	98.00 ± 0.60	98.10 ± 0.64	99.26 ± 0.85	98.77 ± 0.82

^x^ Marketed by: Vedim Sp. z o.o., Warszawa, Poland; ^y^ Average of three determinations; ^z^ n.d.—not determined
